# Deep learning-based segmentation of brain parenchyma and ventricular system in CT scans in the presence of anomalies

**DOI:** 10.3389/fnimg.2023.1228255

**Published:** 2023-08-04

**Authors:** Annika Gerken, Sina Walluscheck, Peter Kohlmann, Ivana Galinovic, Kersten Villringer, Jochen B. Fiebach, Jan Klein, Stefan Heldmann

**Affiliations:** ^1^Fraunhofer Institute for Digital Medicine MEVIS, Bremen, Germany; ^2^Fraunhofer Institute for Digital Medicine MEVIS, Lübeck, Germany; ^3^Fraunhofer Institute for Digital Medicine MEVIS, Berlin, Germany; ^4^Center for Stroke Research Berlin (CSB) Charité, Universitätsmedizin, Berlin, Berlin, Germany

**Keywords:** deep learning, segmentation, hemorrhage, parenchyma, ventricular system

## Abstract

**Introduction:**

The automatic segmentation of brain parenchyma and cerebrospinal fluid-filled spaces such as the ventricular system is the first step for quantitative and qualitative analysis of brain CT data. For clinical practice and especially for diagnostics, it is crucial that such a method is robust to anatomical variability and pathological changes such as (hemorrhagic or neoplastic) lesions and chronic defects. This study investigates the increase in overall robustness of a deep learning algorithm that is gained by adding hemorrhage training data to an otherwise normal training cohort.

**Methods:**

A 2D U-Net is trained on subjects with normal appearing brain anatomy. In a second experiment the training data includes additional subjects with brain hemorrhage on image data of the RSNA Brain CT Hemorrhage Challenge with custom reference segmentations. The resulting networks are evaluated on normal and hemorrhage test casesseparately, and on an independent test set of patients with brain tumors of the publicly available GLIS-RT dataset.

**Results:**

Adding data with hemorrhage to the training set significantly improves the segmentation performance over an algorithm trained exclusively on normally appearing data, not only in the hemorrhage test set but also in the tumor test set. The performance on normally appearing data is stable. Overall, the improved algorithm achieves median Dice scores of 0.98 (parenchyma), 0.91 (left ventricle), 0.90 (right ventricle), 0.81 (third ventricle), and 0.80 (fourth ventricle) on the hemorrhage test set. On the tumor test set, the median Dice scores are 0.96 (parenchyma), 0.90 (left ventricle), 0.90 (right ventricle), 0.75 (third ventricle), and 0.73 (fourth ventricle).

**Conclusion:**

Training on an extended data set that includes pathologies is crucial and significantly increases the overall robustness of a segmentation algorithm for brain parenchyma and ventricular system in CT data, also for anomalies completely unseen during training. Extension of the training set to include other diseases may further improve the generalizability of the algorithm.

## 1. Introduction

Computed tomography (CT) is a common radiological diagnostic tool to assess the brain for anomalies such as stroke, hemorrhagic or neoplastic lesions as well as other structural changes, as it combines high spatial resolution with good image contrast to surrounding tissue. Moreover, changes in the general brain anatomy such as shape or volume of the brain parenchyma and ventricular system are often hints to an underlying pathological condition. After preprocessing of data (conversion of DICOM to other formats, brain extraction and image registration), successful segmentation of basic structures such as brain parenchyma and spaces filled with cerebrospinal fluid (CSF), including the ventricular system, constitutes the base prerequisite for further tasks of identifying and correctly classifying particular intracranial pathologies. Here it is of singular importance that the algorithm be attuned to “real world” data, which incorporates the wide spectrum of anatomical variants as well as frequently encountered pathological conditions such as chronic defects and hemorrhagic or neoplastic lesions.

Due to the very high contrast of the ventricles to surrounding brain tissue, a number of automatic segmentation methods of ventricles in CT data have been proposed that rely on prior anatomical knowledge or explicit modeling. This includes solutions using template or atlas matching (Chen et al., 2009; Poh et al., 2012; Vos et al., 2013), threshold optimization (Qian et al., 2017), or level set methods (Jayaraman et al., 2020). However, unless explicitly modeled, such handcrafted solutions may fail in the presence of anomalies that lead to intensity or morphological changes within or adjacent to the ventricles such as hemorrhage or stroke lesions.

Through the implementation of deep learning (DL) technologies, society hopes to improve the accuracy, speed, and standardization of neuroimaging diagnosis (Yeo et al., 2021), thus supporting the physician, particularly in low throughput centers and during nights or weekends when there might be reduced availability of on-call radiologists. Multiple DL-based methods for segmentation of brain parenchyma and ventricles have been proposed: either for direct quantification (Huff et al., 2019; Zhou et al., 2020) or as a pre-processing step for registration purposes (Dubost et al., 2020; Walluscheck et al., 2023). In further studies, substructures of the brain parenchyma are segmented (Cai et al., 2020; Zopes et al., 2021) or a cross-modality approach for CT and MRI is proposed (Zopes et al., 2021; Zhou et al., 2022). However, these studies often focus on specific diseases such as hydrocephalus or are evaluated only on data of healthy or elderly patients. Under these circumstances, a good generalization and high robustness to brain changes in patients with other diseases and comorbidities cannot be assumed.

In this work, we show that a DL-based solution for the segmentation of brain parenchyma and ventricular system, trained exclusively on patients with normal anatomy (i.e. no apparent structural changes or lesions), does not generalize robustly to patients with brain anomalies, and that extending the training set to a specific type of anomaly (hemorrhage) also improves the robustness in the case of other anomalies (tumors).

## 2. Methods

In this section we will give details on the used training and test data (Section 2.1 as well as the training process and model (see Section 2.2). We will shortly introduce how our model was evaluated in section Section 2.3.

### 2.1. Data

For this study, only publicly available CT data was used, some with custom reference segmentations as described below.

#### 2.1.1. RSNA 2019 brain CT hemorrhage challenge

We use different subsets of the RSNA 2019 Brain CT Hemorrhage Challenge (Flanders et al., 2020) to create multiple test and training data sets. First, we divided the whole data set into: (1) a “normal” cohort of 220 subjects without hemorrhage or other obvious anomalies and (2) an “anomaly” cohort of 426 subjects, all with brain hemorrhage (epidural, intraparenchymal, intraventricular, subarachnoid or subdural).

In the normal cohort, five target structures were annotated fully manually by radiographers and verified by a radiologist: brain parenchyma and ventricular system composed of left ventricle, right ventricle, third and fourth ventricle. These data were split into 158 cases for training, 40 cases for validation during training, and 22 cases for testing. We refer to this data as “normal” test/training data.

To generate training/testing segmentation mask for the anomaly cohort, an nnU-Net (Isensee et al., 2021) was trained on the normal image cohort, generating pre-segmentations for the anomaly cohort. The total number of pre-segmentations was split between two radiologists (IG, KV; each well over a decade of experience in neuroimaging) who manually corrected their cases respectively. In cases of uncertainty, a neuroradiologist (JF) was consulted. A subset of 124 cases was annotated by both readers, resulting in two structure contours per case. If assigned to the training or validation set, both contour variants were used separately. If assigned to the test set, the two individual segmentations were merged by union to simplify the evaluation. In some cases where the pre-segmentation was of very poor quality, only a subset of the five target structures was segmented. Only in the test set, all structures were corrected in all cases. The data were split into 334 individual subjects for training, 22 subjects for validation, and 70 subjects for testing. Table 1 gives an overview of the number of annotated structures per data subset. All uncorrected structures were excluded from training and validation as described in Section 2.2. In the course of this paper we refer to the anomaly cohort of the RSNA data as “hemorrhage data.”

**Table 1:** Number of annotated structures in normal and hemorrhage data sets for parenchyma (P), left ventricle (LV), right ventricle (RV), third ventricle (3rdV), and fourth ventricle (4thV).

	**Normal cohort**	**Anomaly cohort**				
**Structures**	**All**	**P**	**LV**	**RV**	**3rdV**	**4thV**
Training	158	289	261	268	337	362
Validation	40	27	20	20	30	30
Test	22	70	70	70	70	70

#### 2.1.2. Glioma Image Segmentation for Radiotherapy

For additional validation on independent data with a different kind of anomaly than hemorrhage, a subset of the Glioma Image Segmentation for Radiotherapy (GLIS-RT) collection (Shusharina and Bortfeld, 2021; Shusharina et al., 2021) was used, available via The Cancer Imaging Archive (TCIA) (Clark et al., 2013). The dataset contains patients with glioblastoma or low-grade glioma, with CT scans acquired for radiotherapy planning after diagnostic imaging. Not all cases contain reference contours of all structures of interest, therefore only a subset of 106 patients with contours of the brain parenchyma and individual ventricular structures was used for testing. To differentiate between this anomalous test set and the previously described “hemorrhage data” we will refer to the GLIS-RT test set as “glioma data”.

#### 2.1.3. Data pre-processing

The images were resampled in the axial plane only to a range of 0.4–0.6 mm, without resampling between the individual (thick) slices. Any images with lower/higher resolution were resampled to the upper/lower bound of this range, all other images were not resampled. The Hounsfield units were clipped to a subdural window range of (–n20 HU, 180 HU). For data augmentation, flipping of left and right body side was applied during training, where reference labels for left and right ventricle were swapped whenever an image was flipped.

### 2.2. Automatic segmentation

Segmentation is performed by deep Learning using a common U-Net architecture (Ronneberger et al., 2015). Our 3D training data consists mostly of axial slices with fine in-plane resolution below 1 mm and 5mm thickness. For this reason, we conducted preliminary experiments using a 3D and a 2D segmentation model, specifically the 3D aU-Net (Chlebus et al., 2022) designed to process anisotropic data. In our experiments, we compared the performance of the 2D and 3D approaches on normal data. The results are shown in Table 2. The accuracy of the two approaches is essentially identical, which is most likely due to the very high slice thickness of 5 mm in the RSNA dataset. However, the runtime of the 3D approach was significantly longer, so we decided to use the 2D U-Net as the basis for our work.

**Table 2:** Mean dice and standard deviation for two network architectures trained on normal data for parenchyma (P), left ventricle (LV), right ventricle (RV), third ventricle (3rdV), and fourth ventricle (4thV).

	**Runtime**	**Dice**				
**Network**	**Per dataset**	**P**	**LV**	**RV**	**3rdV**	**4thV**
3D aU-Net	18.8 s	0.96 ± 0.02	0.86 ± 0.06	0.85 ± 0.06	0.77 ± 0.07	0.81 ± 0.06
2D U-Net	3.8 s	0.96 ± 0.02	0.88 ± 0.05	0.87 ± 0.06	0.76 ± 0.09	0.81 ± 0.06

Evaluation done on validation split for model selection.

For regularization, dropout (*p* = 0.25) (Srivastava et al., 2014) and batch normalization (Ioffe and Szegedy, 2015) were used. PReLU (He et al., 2015) was chosen as activation function. The loss function and optimization were chosen following the nnU-Net framework: the combined Dice and categorical crossentropy loss were optimized using stochastic gradient descent with Nesterov momentum 0.99 with a polynomial learning rate decay scheme. The training ran for 125,000 iterations with batch size 10 and patch size 260 × 260 at the model output, with a validation step each 500 iterations. The final model was chosen based on the highest mean validation Jaccard score across all five target structures.

For training with incomplete annotations in the case of the hemorrhage data, a weighting scheme was implemented that excludes all missing structures (and in case of incomplete annotation also the background channel) from the loss calculation (Petit et al., 2018).

The 2D U-Net was trained twice: (1) on the normal cohort only and (2) on the joint normal and hemorrhage data. In both trainings, the sampling of patches was adjusted so that all five structures of interest were sampled with similar frequencies, to account for large size differences and class imbalance of parenchyma and ventricular structures. In training (1), patches including foreground voxels of any of the five structures vs. pure background patches were sampled in a ratio of 4:1. In training (2), the ratio was 9:1, with four foreground patches from the normal cohort and five foreground patches from the hemorrhage data with one patch from each of the available hemorrhage classes (epidural, intraparenchymal, intraventricular, subarachnoid, subdural) to enable learning of the different anomalies.

For post-processing, the largest connected component of the predicted parenchyma mask was extracted. The ventricular structures were then restricted to the convex hull of the parenchyma mask, to remove any false positives outside the brain region. No further connected component analysis was performed for the ventricles, due to the high slice thickness. In cases with fine connections of the ventricle's body to the temporal horn, or separate annotated components of the third ventricle, this would remove part of the valid predicted structure. However, in few cases this might lead to small, distant false positives and therefore outliers in calculation of the Hausdorff distance metric.

### 2.3. Evaluation

Both trained networks were evaluated separately on the following three test sets: (1) 22 test cases of the normal RSNA cohort, (2) 70 test cases of the RSNA hemorrhage data, and (3) 106 cases of the glioma dataset. For all evaluation experiments we report the Dice score and 95%-Hausdorff distance. Calculation of statistical significance with correction for multiple testing (per metric and test set) using the Benjamini-Hochberg procedure was performed with the statannotation python package (Charlier et al., 2022).

## 3. Results

In the following sections, we present results on the normal and hemorrhage test cases of the RSNA Brain CT Hemorrhage challenge (Section 3.1) and on the glioma test set (Section 3.2).

### 3.1. RSNA (normal and hemorrhage) test sets

**Figure 1:**
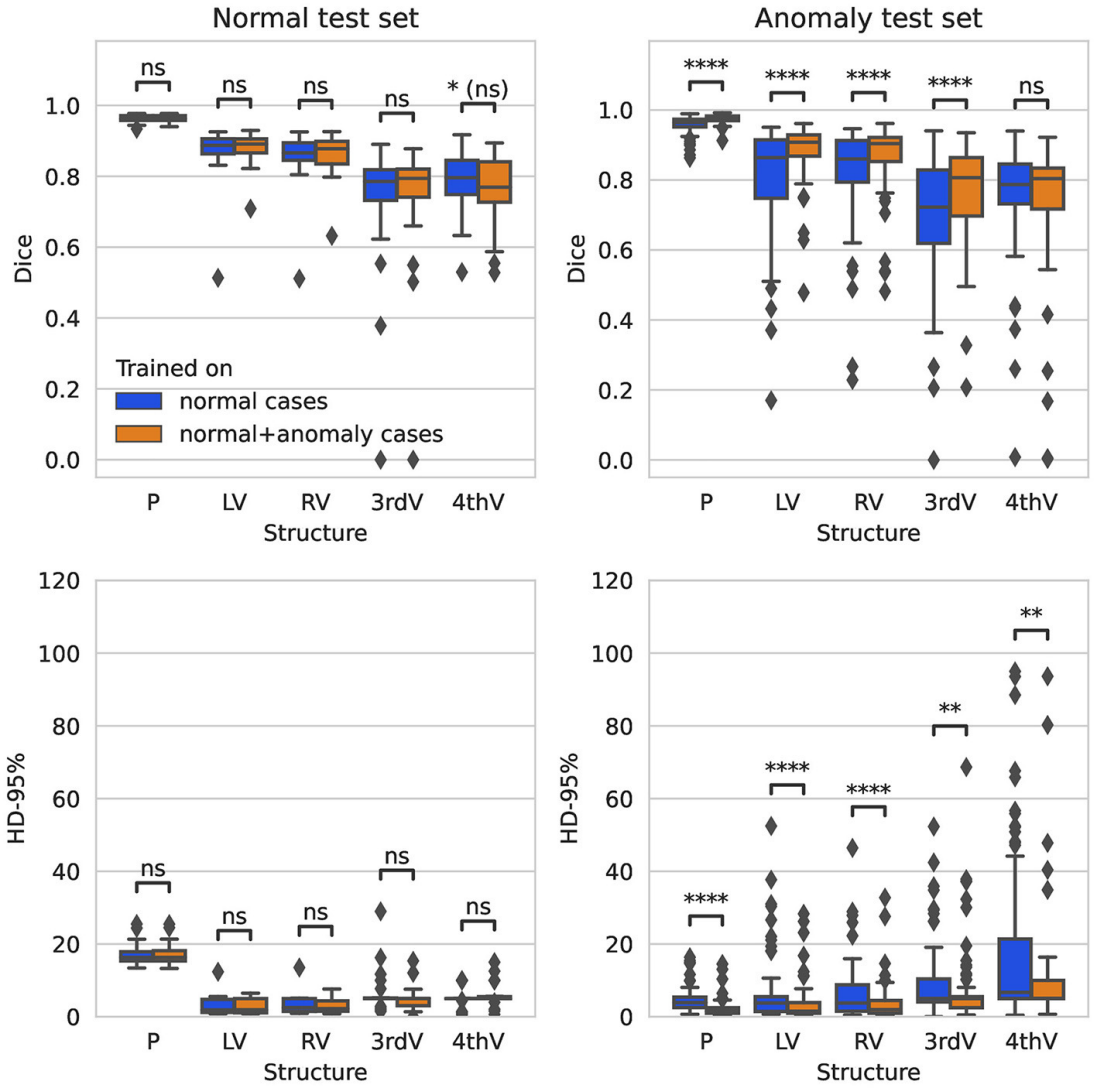
Boxplot of Dice scores and 95%-Hausdorff distance for the segmentation of parenchyma (P), left ventricle (LV), right ventricle (RV), third ventricle (3rdV), and fourth ventricle (4thV) using the two U-Nets trained on normal only vs. normal plus hemorrhage data on normal and hemorrhage test sets.

On the normal test set, both trained networks show no significant difference in performance after correction for multiple testing for all five target structures for Dice and 95%-Hausdorff distance metrics, see Figure 1. On the hemorrhage test set, the Dice score is increased significantly for all structures with the exception of fourth ventricle, when including anomaly data into the training. The 95%-Hausdorff metric is significantly reduced for all structures. On average, the Dice score of the improved model on the hemorrhage test set is similar compared to the normal test set for all structures. The 95%-Hausdorff distance has more severe outliers on the anomaly test set, due to small but distant false positives as described in Section 2.2.

**Figure 2:**
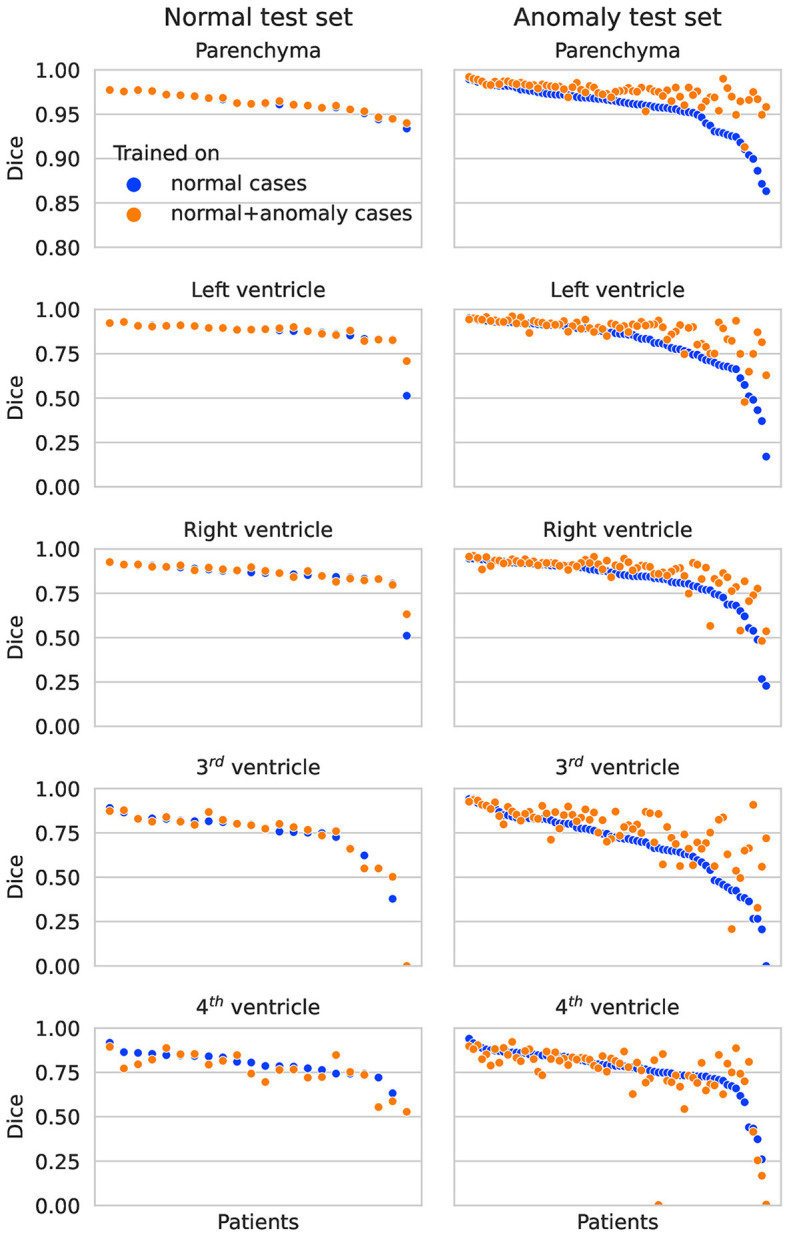
Dice scores per patient on the normal test set **(left column)** and anomaly test set **(right column)** for each structure. The two colors represent results for the network trained on normal cases only vs. normal plus hemorrhage cases.

In Figure 2, the Dice scores achieved by both networks are plotted per test subject in descending order. On the normal test set, the performance of both networks is almost identical for all subjects. On the hemorrhage test set, the performance of the network trained exclusively on normal cases drops considerably for several subjects, whereas the network also trained on anomaly cases is more robust and maintains an overall higher performance. This direct per case comparison illustrates well that the segmentation performance of the model trained on additional hemorrhage data drops in only few cases, again with the exception of the fourth ventricle.

**Figure 3:**
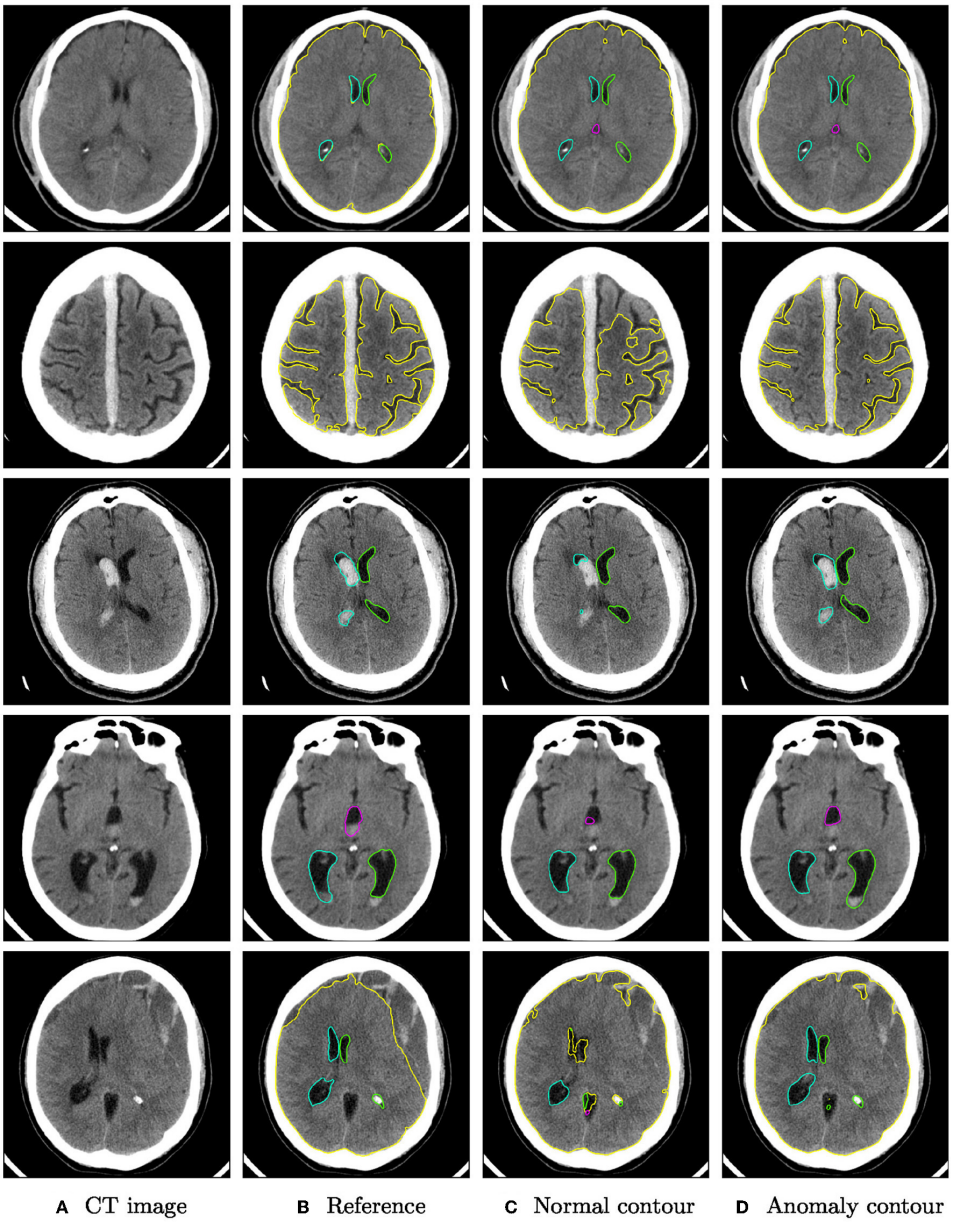
Qualitative segmentation examples from the RSNA dataset of parenchyma (yellow) and ventricles (left ventricle: green, right ventricle: cyan, third ventricle: magenta, fourth ventricle: purple). Each row shows from left to right **(A)** the original CT slices, **(B)** the reference segmentation, **(C)** the segmentation from the algorithm trained on normal cases only, and **(D)** the segmentation from the algorithm trained on normal cases and hemorrhage cases. For better visibility, not all structures are shown for all cases.

Qualitative segmentation results of both networks are shown in Figure 3 on anomaly cases with different hemorrhage classes. In cases with small anomalies (row 1: small subdural bleed on right hemisphere), both networks achieve similar results. In cases with larger anomalies (row 2: subdural bleed between hemispheres, row 3: intraventricular bleed, row 4: intraventricular bleed), the network trained also on hemorrhage cases is much more robust. However, some anomalies may still be missed, such as smaller intraventricular bleeds with lower HU values. In some cases (row 5: large mostly iso- or hypodense subdural bleed), both networks fail in a similar way for parenchyma segmentation.

### 3.2. GLIS-RT (tumor) test set

**Figure 4:**
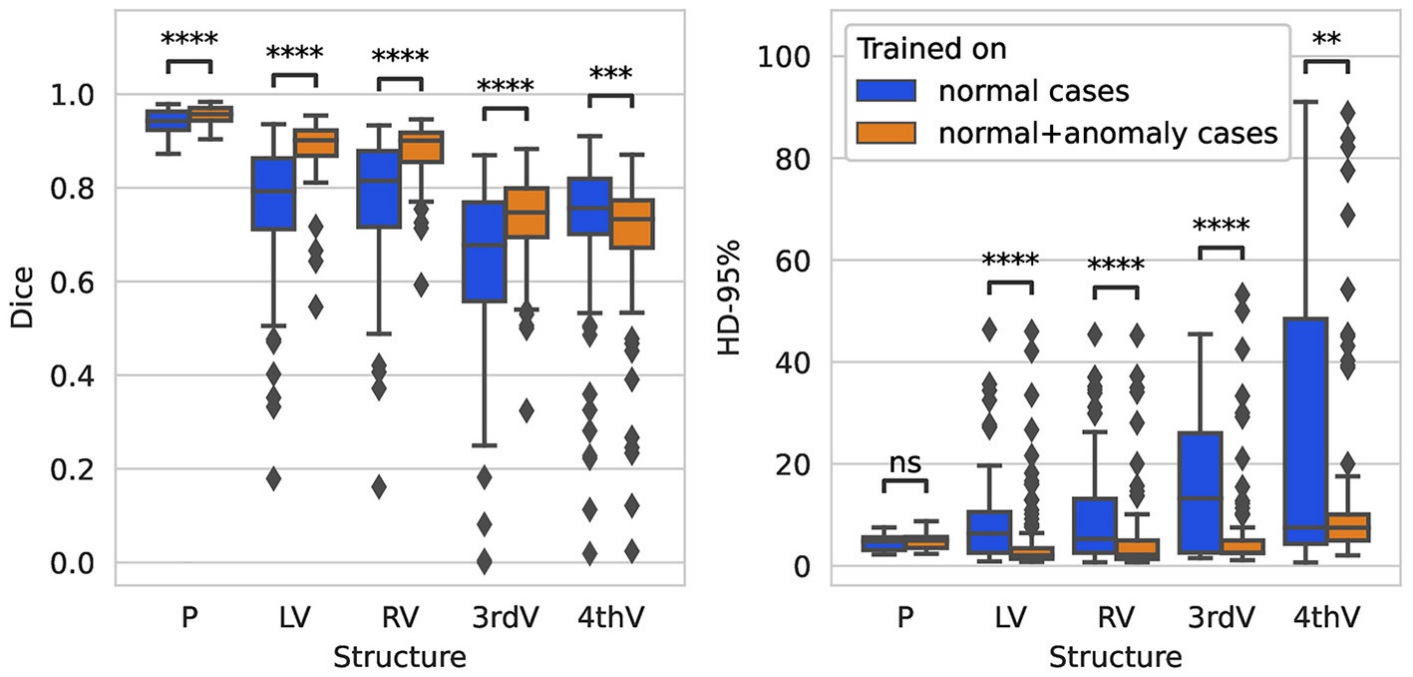
Boxplot of Dice scores and 95%-Hausdorff distance for the segmentation of parenchyma (P), left ventricle (LV), right ventricle (RV), third ventricle (3rdV), and fourth ventricle (4thV) using the two U-Nets trained on normal only vs. normal plus hemorrhage data on the glioma test set.

On the glioma test set, the segmentation performance measured by the Dice score is significantly improved for all structures when adding anomaly (hemorrhage) data to the training, see Figure 4. The 95%-Hausdorff distance is significantly reduced for all structures except for parenchyma, where it is already low for the U-Net trained only on normal data. As shown in Table 3, the median Dice score of the improved model on the glioma data is similar to the RSNA data for parenchyma and lateral ventricles, but slightly reduced for third and fourth ventricle. The median 95%-Hausdorff distance is slightly lower for the third ventricle, but higher for the fourth ventricle, for parenchyma and lateral ventricles it is similar.

**Table 3:** Median (and inter-quartile range) of dice score and 95%-Hausdorff distance for the U-Net trained on normal and hemorrhage data on the three different test sets.

		**Test set**		
**Metric**	**Structure**	**RSNA normal**	**RSNA anomaly**	**GLIS-RT**
	Parenchyma	0.96 (0.96–0.97)	0.98 (0.97–0.98)	0.96 (0.94–0.97)
	Left ventricle	0.89 (0.87–0.91)	0.91 (0.87–0.93)	0.90 (0.87–0.92)
Dice	Right ventricle	0.88 (0.83–0.90)	0.90 (0.85–0.92)	0.90 (0.86–0.92)
	3^rd^ ventricle	0.79 (0.74–0.82)	0.81 (0.70–0.86)	0.75 (0.69–0.80)
	4^th^ ventricle	0.77 (0.73–0.84)	0.80 (0.72–0.83)	0.73 (0.67–0.77)
	Parenchyma	16.33 (15.36–18.18)	1.54 (0.98–2.50)	5.00 (3.52–5.65)
	Left ventricle	1.92 (1.09–5.00)	1.54 (0.98–3.89)	2.05 (1.37–3.46)
HD-95%	Right ventricle	2.22 (1.51–4.39)	1.95 (0.98–4.49)	2.21 (1.36–5.00)
	3^rd^ ventricle	5.00 (3.03–5.02)	5.00 (2.50–5.52)	2.59 (2.50–5.00)
	4^th^ ventricle	5.01 (5.00–5.50)	5.04 (5.00–10.00)	7.50 (5.00–10.08)

**Figure 5:**
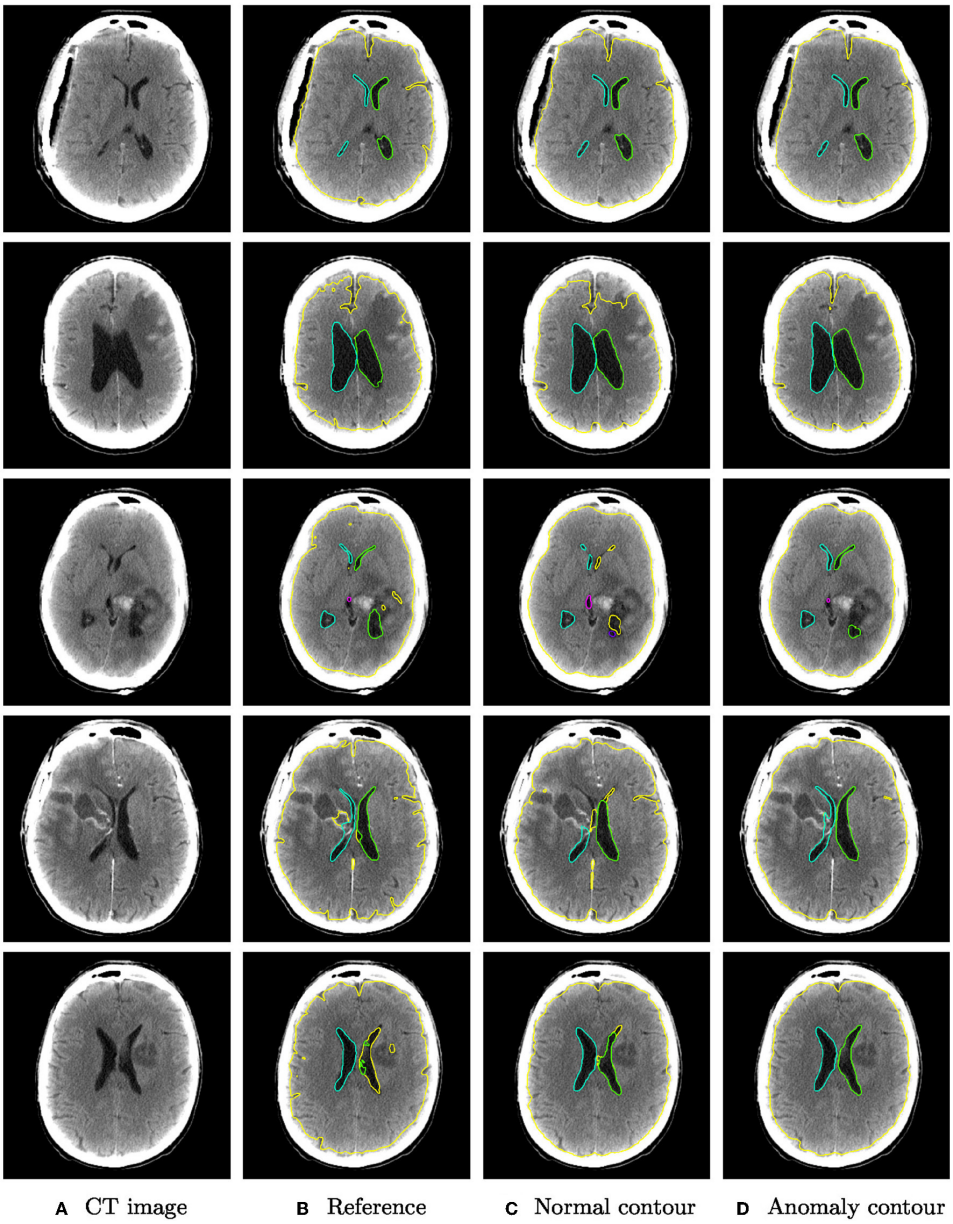
Qualitative segmentation examples from the glioma dataset of parenchyma including tumors (yellow) and ventricles (left ventricle: green, right ventricle: cyan, third ventricle: magenta, fourth ventricle: purple). Each row shows from left to right **(A)** the original CT slices, **(B)** the reference segmentation, **(C)** the segmentation from the algorithm trained on normal cases only, and **(D)** the segmentation from the algorithm trained on normal cases and hemorrhage cases.

Figure 5 shows qualitative examples from the glioma dataset. The algorithm trained also on anomaly data is overall more robust to changes in intensity than the algorithm trained only on normal data. In some cases, the improved segmentation even outperforms the reference segmentation (rows 3–5).

## 4. Discussion

The field of DL-based quantitative neuroimage analysis is rapidly expanding and currently already covers commercial applications for non-contrast CT to identify acute ischemia, arterial obstruction seen as hyperattenuated arteries, brain hemorrhage or brain trauma. However, there has been much criticism of the certification process for artificial intelligence (AI) software in the radiological community (Wardlaw et al., 2022). Currently such software can be marketed in the European Economic Area (EEA) after achieving a Conformité Europëenne (CE) mark but the standards for achieving such a certification are surprisingly low. Exploiting the loophole created by the phrasing that they are designed to only support (not replace) medical decision making, AI software for radiology is often awarded the CE mark without external scrutiny. An independent review of all CE-marked AI software for radiology in Europe published in 2021 found that 64 of 100 products had no published peer-reviewed evidence of efficacy (van Leeuwen et al., 2021). Therefore, we chose to perform a study that focuses on the training and validation data and to develop an algorithm that is robust to unseen pathologies in the input data, rather than focus on methodological improvements of the architecture or training process.

With this aim, we have demonstrated that a robust and fully automatic segmentation of brain parenchyma and ventricular system can be achieved by deep learning. Unlike previous publications on the topic, that have either excluded subjects with severe anomalies (e.g., Huff et al., 2019; Cai et al., 2020) or focused on a particular kind of disease (e.g., Zhou et al., 2020, 2022), our developed algorithm is robust for two different kinds of anomalies (intracranial hemorrhage and tumors), despite being trained only on normal and hemorrhage data with partially incomplete annotations. In addition, the higher generalizability and robustness to anatomical changes does not come with a decrease in segmentation performance on normal anatomy, which is equally important. The resulting algorithm can therefore likely be applied in a wide range of clinical scenarios.

In this initial study, the evaluation of the developed algorithm was still limited to two kinds of anomalies and should be validated on a larger test set, including overall more subjects and more diverse anatomical changes. Moreover, the training set should be extended to better reflect the diversity of possible anomalies to further increase the robustness. A major obstacle in this is the effort for manually curated ground truth data. The robust segmentation algorithm could serve as an automatic pre-segmentation step that will likely reduce the time required for manual corrections in an iterative workflow.

Future work should extend the evaluation to clinically used parameters such as volumetric measurements, in addition to common segmentation measures like the Dice score and Hausdorff distance. Testing the model on a larger data set with different imaging or patient properties could add further insights on the generalizability of the model. In the future, possibilities for integrating the method into clinical workflow shall be explored.

## Data availability statement

The datasets presented in this article are not readily available because, custom reference segmentations have been created. The image data of the RSNA 2019 Brain CT Hemorrhage and Glioma Image Segmentation for Radiotherapy (GLIS-RT) collection are publicly available. Requests to access the datasets should be directed at: SH, stefan.heldmann@mevis.fraunhofer.de.

## Author contributions

AG, SW, JK, and SH contributed to conception and design of the study. AG and PK organized the database. IG, KV, and JF annotated the hemorrhage anomaly data set. AG implemented methods, performed experiments and the statistical analysis, and wrote the first draft of the manuscript. AG and IG wrote sections of the manuscript. All authors contributed to manuscript revision, read, and approved the submitted version.
